# An Architecture for Collaborative Terrain Sketching with Mobile Devices

**DOI:** 10.3390/s21237881

**Published:** 2021-11-26

**Authors:** Sonia Mendoza, Andrés Cortés-Dávalos, Luis Martín Sánchez-Adame, Dominique Decouchant

**Affiliations:** 1 Computer Science Department, CINVESTAV-IPN, Mexico City 07360, Mexico; acortes@computacion.cs.cinvestav.mx (A.C.-D.); luismartin.sanchez@cinvestav.mx (L.M.S.-A.); 2 Information Technologies Department, UAM-Cuajimalpa, Mexico City 05348, Mexico; decouchant@correo.cua.uam.mx; 3 Centre National de la Recherche Scientifique (C.N.R.S.), Laboratoire LIG, University of Grenoble, 38058 Grenoble, France

**Keywords:** co-located collaboration settings, sketching applications, terrain modeling architectures, augmented reality, novice users, heterogeneous mobile devices

## Abstract

3D terrains used in digital animations and videogames are typically created by several collaborators with a single-user application, which constrains them to update the shared terrain from their PCs, using a turn-taking strategy. Moreover, collaborators have to visualize the terrain through 2D views, confusing novice users when conceiving its shape in 3D. In this article, we describe an architecture for collaborative applications, which allow co-located users to sketch a terrain using their mobile devices concurrently. Two interaction modes are supplied: the standard one and an augmented reality-based mode, which helps collaborators understand the 3D terrain shape. Using the painting with brushesparadigm, users can modify the terrain while visualizing its shape evolution through the camera of their devices. Work coordination is promoted by enriching the 3D space with each collaborator’s avatar, which provides awareness information about identity, location, and current action. We implemented a collaborative application from this architecture that was tested by groups of users, who assessed its hedonic and pragmatic qualities in both interaction modes and compared them with the qualities of a similar Web terrain editor. The results showed that the augmented reality mode of our prototype was considered more attractive and usable by the participants.

## 1. Introduction

Augmented Reality (AR) is not a new topic, but a significant increase in its popularity has been seen in recent years. This may be because AR has improved a lot [1]. After all, if starting from the premise that the main objective of AR is to integrate the real world with the digital one, new technologies have allowed users to have more complex and positive experiences that comply with this goal [2]. This is a consequence of the fact that multiple developers have focused their efforts on integrating AR with mobile devices, since they offer considerable computing power with the advantages of being portable and affordable for many people [3].

Mobile devices have allowed work contexts to be transformed into highly interactive environments, changing our way of viewing information and interacting with other people, creating experiences beyond the classic scenario of a single-user with a single-computer [3]. However, this technological democratization also poses serious problems, e.g., complex systems (like 3D modeling software) are typically used by people with advanced knowledge, or experts familiar with a particular way of working and handling data. This imposes an entry barrier for people involved in solving a complex problem, so it is essential to facilitate the understanding of these systems by people with varying degrees of experience. Interactions between people in everyday environments should also be eased [3].

Numerous 3D modeling applications can be found in the commercial sector and free software community, but most of them present problems for novice users, typically with a steep learning curve [4]. Their user interface usually has many editing tools and complicated options, and the interaction can be complex, since these applications rely on the traditional 3D interaction paradigm: the input is provided by 2D peripherals, such as mouse and keyboard, and the 3D model has to be visualized through 2D views taken from different virtual cameras [5]. However, novice users are not wholly aware of these cameras, so they encounter difficulties turning a camera towards the intended position or switching it [6]. In addition, amateur users cannot quickly figure out the aspect of the 3D model just from 2D views, which can suddenly disappear in the 3D virtual space.

On the other hand, these applications are single-user, so they obviously lack adequate support for group work [7]. Thus, collaboration is usually limited to asynchronous settings, in which 3D models are sequentially built by several persons using a single-user application. In this case, they are normally affixed to static computers and coordinate their activities without any support from the application [8]. Only one person works on the 3D model during a collaborative session, while the other group members have to wait or observe [9]. Later, other collaborators can suggest changes to the colleague that is interacting with the application; at times, they can take turns in order to perform their own part of the shared work. Obviously, the applications neither support simultaneous interaction with multiple collaborators nor maintain shared data consistency. It is clear that, in some steps of group work, it is more efficient to have several persons producing simultaneously than sequentially [10].

These problems are the motivation for our proposal, and to give it greater clarity, a real-life scenario is introduced: a group of colleagues is discussing ideas about the shape of a surface intended for a video game. They do not have a PC nearby, but each one carries a smartphone or tablet. Instead of trying to convey their ideas just by speaking, gesticulating, or sketching on a napkin, making room for lots of misunderstandings, they could sketch such a surface with a collaborative application from their mobile devices, using technologies, like AR, that facilitate this common task [11]. Once finished with the first version of their sketch, these colleagues can save it in a format understandable by a 3D modeling application. Later, that sketch can be imported, for instance, in CoMaya [12,13] serving as a basis to model a 3D object with colors, textures, and movements (or behavior) using the sophisticated tools provided by the aforementioned application.

As a solution for these problems, ShAREdT ( Shared Augmented Reality-based Editing for Terrains) is proposed in this article. It is a novel architecture for developing collaborative applications that facilitate 3D terrain sketches by a group of co-located people, using AR technology on their mobile devices with no extra equipment (e.g., stereoscopic viewers, Kinects, projectors, LiDAR, or Microsoft HoloLens devices). The target users of the applications developed with our proposed architecture are collaborators who may not have prior training on 3D modeling and experts who might need to sketch basic terrains on the go.

We built a prototype following our architecture’s guidelines, a collaborative mobile application that allows basic 3D sketching of terrains, where users can model by visualizing the terrain solely through the display of their device or with AR. For testing, groups of novice users in 3D modeling have evaluated the hedonic and pragmatic qualities of this prototype and have compared it with a similar application: WebGL Terrain Editor [14]. The results show that our proposal can improve the user experience of 3D terrain sketching for beginners.

This article is organized in the following manner. After presenting related work on terrain modeling applications in Section 2, the next main components of the ShAREdT architecture are explained in Section 3: standard and AR-based interaction modes, terrain modeling, 3D scene management, group awareness, and data sharing. Implementation details of the collaborative application for terrain sketching that has been developed to validate the ShAREdT architecture are given in Section 4, where we describe the three types of widgets that provide virtual objects with desired behaviors in the 3D scene. Then, Section 5 presents the tests conducted with users of our new prototype, and Section 6 states the contributions, future extensions, and limitations of the achieved work. Finally, a conclusion is provided in Section 7.

## 2. Related Work

**Figure 1:**
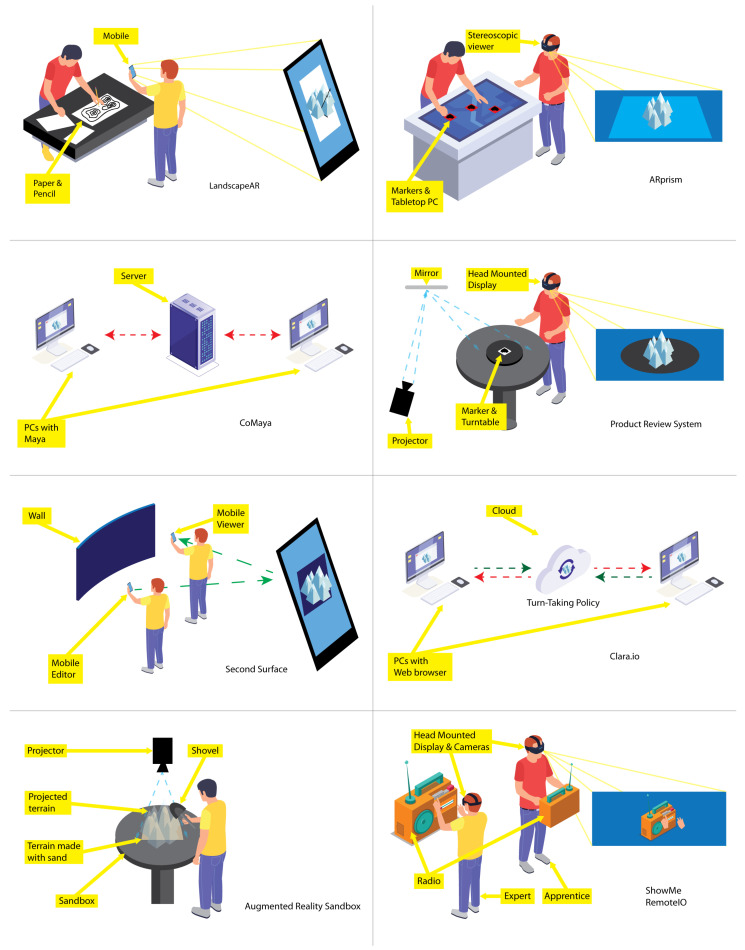
Different approaches for terrain construction and AR-based collaboration.

In the following paragraphs, relevant terrain sketching applications are analyzed (see Figure 1).

*LandscapAR* [15] is a single-user application for mobile devices that recognizes and tracks an elevation map depicted on a white sheet of paper. The application captures an image of the drawing and generates the elevation map from the level curves. The graphical representation of the terrain is shown on the paper using AR. However, these models may not be altered directly. If some change on the terrain is needed, the user must physically edit the drawing or create a new one, and then the application will generate the new terrain.

*ARprism* [16] is a desktop application that facilitates co-located collaboration for geographical data visualization activities performed by two users, each using a lightweight stereoscopic viewer. This application uses AR technology to add different topographical data layers (e.g., elevation, hydrology, and soil) on a tabletop map. Nevertheless, the 3D terrain representations must be modeled in advance, and the users cannot modify them.

*CoMaya* [12,13] is a collaborative plug-in for Maya [17], that allows several users connected to a central server to work on the same 3D scene concurrently. CoMaya uses the operational transformation technique [18,19] to maintain shared data consistency among the distributed copies. However, transformation rules were created only for a subset of operations. Thus, in case it is not possible to solve conflicting operations, users have to figure them out.

Nam and Sakong [20] developed an AR-based collaborative synchronous workspace for distributed product reviewing that allows a pair of users to analyze an everyday 3D object. This system relies on tangible synchronized turntables and virtual shadows to provide remote collaborators with awareness information. However, users had difficulty pointing in the 3D space through the virtual shadows.

*Second Surface* [21] is a mobile multi-user AR application through which a user can create and place digital contents in the neighboring space of ordinary objects, e.g., lampposts and cars, considered as markers. The user can share those contents but not edit them in real-time with other co-located users. Therefore, this application does not offer the same degree of collaborative support as our proposal.

*Clara.io* [22] is a cloud-based tool that allows users to carry out polygonal mesh-based 3D modeling on a web browser. These models can be shared among several registered users that have editing rights. To collaborate, users have to take turns to modify the same mesh. As these meshes are stored on the cloud, users receive updates about the modified models, simulating real-time editions.

*Augmented Reality Sandbox* [23] is a single-user AR system to create physical topography models. It consists of a sandbox that can be physically manipulated, on top of which is added a color-coded elevation map, along with level curves and simulated water. Although it is a great teaching tool, it is a solution that requires a lot of space and equipment.

*ShowMe* [24] is a collaborative system that facilitates immersive gestural communication between users, a local apprentice, and a remote expert. The apprentice uses a HMD (Head Mounted Display) equipped with two cameras that capture his stereoscopic view, which is sent to the expert, who also uses a HMD. The expert has a depth sensor whereby the HMD captures his hands, to be shown on the local apprentice’s 3D scene, using AR. Additionally, the designers of ShowMe propose *RemotIO* [25], whereby the remote expert can manipulate a radio through manual gestures [26]. Hence, the expert can show the apprentice gestures made with his hands and their effect on the radio since it reacts in real-time. This is a work where collaboration stands out. However, it is aimed at expert users and requires highly specialized equipment.

The aforementioned proposals are summarized in Table 1, according to whether they use AR technology and need extra equipment, whether they are groupware systems and the type of collaboration they support, their application domain, whether they allow concurrent work and their execution platform. From this analysis, it can be seen that converging AR and groupware technologies is not a new idea. On the other hand, several efforts have been made at supporting interaction with 3D objects constructed in advance, as well as on creating and sharing 3D contents with other people, without the possibility of performing changes. However, there are no other proposals that allow co-located novice collaborators to create and modify concurrently in situ digital 3D terrains on their mobile devices with no additional equipment required to use AR technology.

## 3. The ShAREdT Architecture

**Figure 2:**
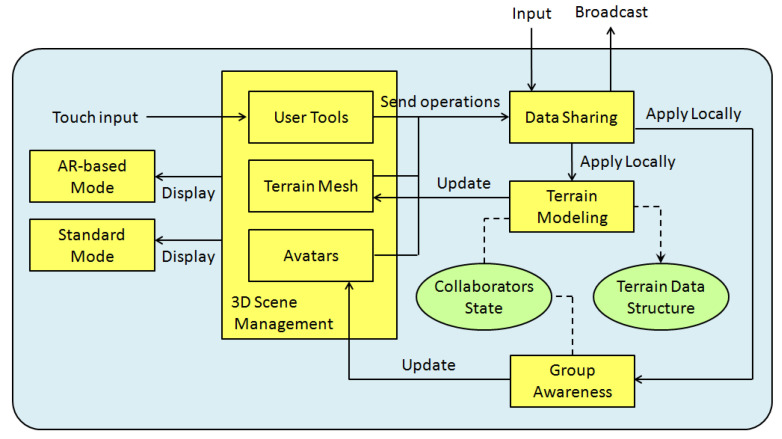
Functional and data components of the ShAREdT architecture.

In this section, we describe the architectural approach to the collaborative modeling of 3D terrains. This architecture is intended to promote face to face collaboration settings, in which participants interact through their mobile devices with the aim of creating and modifying a 3D terrain sketch. Figure 2 shows the main functional and data components of the ShAREdT architecture represented as rectangles and ovals, respectively. Solid arrows denote interactions among functional components; a dotted line means that a functional component manages a data component, and a dotted arrow signifies that a functional component can only consult a data component.

**Figure 3:**
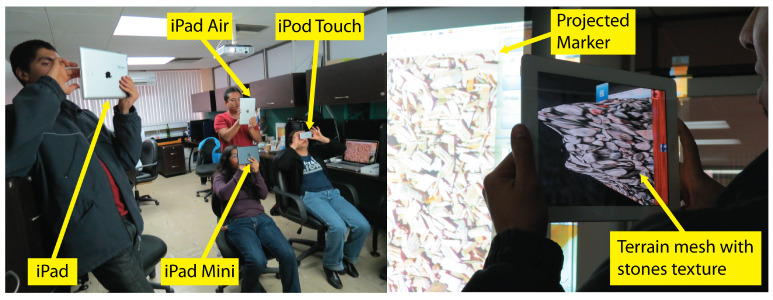
Some participants using the AR mode of our application while the marker is being projected on a wall. The characteristics of the mobile devices are: (1) iPad 3rd generation. Screen size: 9.7 inches. Weight: 660 g, (2) iPad Air 1st generation. Screen size: 9.7 inches. Weight: 469 g, (3) iPad Mini 1st generation. Screen size: 7.9 inches. Weight: 308 g and (4) iPod Touch 5th generation. Screen size: 4 inches. Weight: 88 g. In all devices the camera is 5.0 MP.

In an early stage of our approach, interactions with the 3D scene were just performed using AR over a marker, viewed through the camera of an iPad or iPod (see Figure 3). Using AR in 3D model sketching has several advantages, as mentioned in Section 1. However, from the tests conducted with users of the first version of our prototype [27,28,29], we observed several drawbacks of only using AR to interact with the 3D scene. In some cases, it is convenient to use the collaborative application without AR, thus two interaction modes were included in the ShAREdT architecture: one using AR and the other rendering the 3D scene in the standard way (see the *AR-based Mode* and the *Standard Mode* components in Figure 2).

Another important improvement added to this new iteration of our work is group awareness, a mechanism to inform each user of the actions performed by other group members (see the *Group Awareness* component in Figure 2). In the case of face to face terrain modeling settings, it is not necessary to show a collaborator the viewpoints and positions of other participants, since this awareness information is clearly visible. Instead of this, every collaborator can visualize the terrain mesh points where his colleagues are drawing as well as the kind of operations they are applying. Each collaborator it is shown a small rectangular box which serves as an avatar situated in the terrain mesh coordinates, where the collaborator applied an editing operation, in addition to a label that contains the collaborator’s name, and a sphere that shows the brush properties (see the *Avatars* component in Figure 2).

The *3D Scene Management* component performs the drawing of all objects that have a graphical 3D representation and have to be shown on the screen. Some of these objects are user tool widgets, which are interactable by collaborators (called *User Tools* in Figure 2). Other objects are digital contents, such as the terrain that has a mesh representation (a mesh representation is a particular case of a 3D representation) and is also interactable (called *Terrain Mesh* in Figure 2). Some other objects provide awareness information, such as the aforementioned avatars.

User tool widgets are used to select the editing operation that a collaborator desires to apply. In this case, such widgets do not operate on *Terrain Mesh*, but on *Collaborators State* by sending state change messages to the *Data Sharing* component. These messages are: (1) first broadcasted to other participants, through this component, and (2) then locally processed by the *Group Awareness* component to update *Collaborators State* with the last editing operation selected by the corresponding collaborator.

Likewise, when a collaborator interacts with *Terrain Mesh* by touch events, this component sends editing operation messages to *Data Sharing*. Thus, via this component, the editing operations are transmitted to all users and locally applied on the *Terrain Data Structure* by *Terrain Modeling*, using information provided by *Collaborators State*, such as the selected editing operation, and the brush strength, smoothness, and radius. *Terrain Mesh* is also updated in real-time by the *Terrain Modeling* component.

In the following subsections, several design considerations about the main components of the ShAREdT architecture are described: standard and AR-based interaction modes of the 3D scene (see Section 3.1), terrain modeling (see Section 3.2), 3D scene management (see Section 3.3), awareness information for coordinating group work (see Section 3.4), and data sharing for the concurrent creation and modification of a terrain mesh (see Section 3.5).

### 3.1. Standard and AR-Based Interaction Modes

Human beings perceive reality through five senses: hearing, sight, taste, touch, and smell. In principle, it is possible to cheat our senses by means of artificially generated stimuli, in order to provide a fake perception of virtual objects or environments [30]. Current technology is not sufficient to present full scale virtual environments to all five senses, but there are important advances concerning three of them: sight, hearing, and touch. This is the basis of the Virtual Reality area, whose objective is to immerse the user inside a synthetic world.

**Figure 4:**
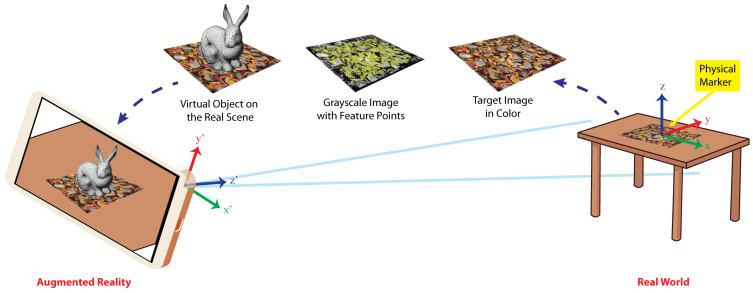
The target color image is taken from the real scene (**right**), then it is transformed to grayscale and its visual feature points in yellow are detected (**center**), and finally a virtual object is added on the real scene (**left**).

A different approach is that of AR, which creates the illusion of virtual objects existing inside the real environment. To achieve AR on mobile devices, the *AR-based Interaction Mode* component relies on effective computer vision and machine learning algorithms to recognize and track markers on which several digital contents can be added. This AR principle is depicted in Figure 4. The scene taken by the camera of a mobile device shows the physical marker placed on the table, which contains a *target image*, i.e., a formerly learned arbitrary image printed on it. Then, the mentioned algorithms transform the target image in greyscale to extract visual feature points on each frame of a video stream.

Using these visual feature points, the *AR-based Interaction Mode* component calculates the transformation matrix $M=R|\vec{t}$, that represents the translation $\vec{t}$ and rotation *R* of the camera with respect to the target image. The matrix *M* transforms points in the reference frame of the target image to points in the reference frame of the camera or conversely; the *Z* axis is also inverted as shown in Figure 4. With the matrix *M*, 3D virtual objects can be drawn correctly aligned with the target image on the video stream. That stream now gives the illusion that the objects are placed inside the real scene. This is shown at the leftmost side of Figure 4. Once the device camera captures the real scene with a marker, the rabbit is drawn at the position and orientation given by the matrix *M*.

We use the Vuforia markers, which gives us the advantage that they can be used virtually (displaying it on a screen or through a projector) or they can be printed on any type of paper and the image can be in color (RGB not CMYK) or in black and white. To capture a marker, there are no orientation restrictions and they can be done from a wide variety of angles. Images for digital markers can be in JPG or PNG formats with a color depth between 8 and 24 bits, with a maximum size of 2.25 MB and a minimum width of 320 pixels.

Using AR technology in 3D modeling applications presents two advantages for users [31]: (1) an enhanced understanding of the 3D shape of virtual objects, and (2) a more natural behavior to control the viewpoint from which the virtual scene is visualized. Nevertheless, the usage of AR also presents some limitations, e.g., in case the mobile device is heavy or large (the 5th generation iPad Pro weights up to 685 g), it might be difficult for the user to hold it with a hand, while depicting on the terrain mesh with the other (see Figure 3).

**Figure 5:**
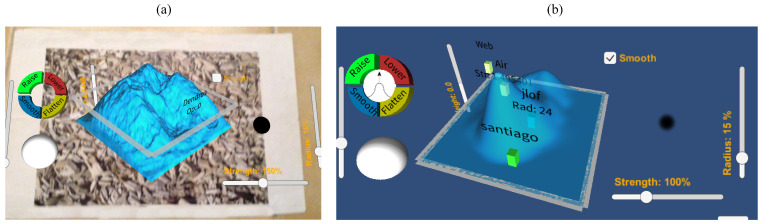
Snapshots of (**a**) the AR-based and (**b**) standard interaction modes.

Taking these concerns into account, two interaction modes were included in the ShAREdT architecture: (1) using the *AR-based Mode* component, where the 3D terrain and widgets are shown to the collaborator with AR over a marker (see the left side of Figure 5), and (2) using the *Standard Mode* component, which allows the user to interact with the 3D scene entirely through the display’s device, i.e., no need of marker nor camera’s device (see right side of Figure 5).

Both modes of interaction are always available to users, and they can switch between them freely. For example, when AR is not required because they are in a tiny place or do not have the marker, they can switch to standard mode. In a collaboration scenario, each user can decide the most convenient mode for him at any moment.

### 3.2. Terrain Modeling Component

The design of the *Terrain Modeling* component is tightly bound to the type of 3D models that the developer needs to manipulate. Thus, this component modifies the terrain data structure and updates the vertices of the terrain mesh, according to the editing operations defined by the 3D model. These abstractions are described in the following two subsections.

#### 3.2.1. Terrain Data Structure

A terrain can be defined as a piece of territory or land containing geometric characteristics that define its shape, e.g., canyons, cracks, craters, lake basins, mountains, plains, riverbeds, and valleys.

**Figure 6:**
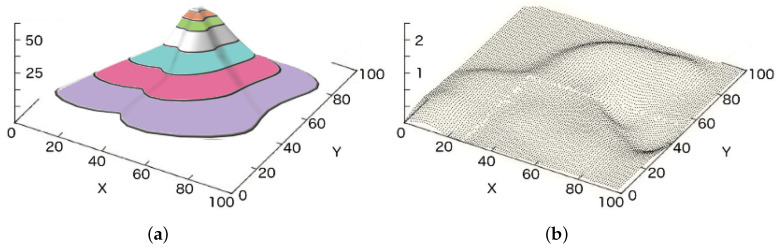
(**a**) The plot of a continuous elevation map with level curves, and (**b**) the plot of a DEM: for every point of the discrete domain, a discrete height value exists.

The most straightforward approach to abstracting the structure of a terrain into a model is giving a height value for every point on its area, where this height value is measured in relation to some accepted reference, e.g., the sea level. A terrain can be described using a continuous function, e.g., Figure 6a shows the plot of a continuous elevation map, in the form of a mountain, with level curves on the 3D surface. However, the ShAREdT architecture makes use of a terrain representation known as Digital Elevation Map (DEM), which is a discrete function:

h:R→F,

where R is the rectangular region $\left[1,m\right]\times \left[1,n\right]\subset N^{2}$ for given integers m,n∈N, and F denotes the domain of floating point numbers that can be saved in a computer. Thus, a 3D terrain is stored as a floating point array of m×n size.

The *h* function cannot describe all aspects of a real terrain, e.g., a layered terrain composition, overhangs, or caves. However, this representation satisfies most of the applications. Figure 6b shows the plot of discrete points that compose a DEM.

In the ShAREdT architecture, a terrain is represented in a graphical way as a 3D surface, employing a polygonal mesh of 100×100 squares.

#### 3.2.2. Brush-Based Operations

To manipulate the shape of a 3D terrain, the *Terrain Modeling* component implements the *painting with brushes* paradigm, which aims at emulating the process of applying paint to an area using a brush. The reader is surely familiar with this paradigm, since it is used in most drawing applications. Indeed, this paradigm is the *de facto* standard for terrain editors, e.g., the *procedural brushes* method [32] and the WebGL terrain editor [14].

Just like a real brush, whereby a person can paint with multiple colors on an area, a collaborator can perform various editing operations on a terrain using a digital brush. The ShAREdT architecture provides support for four elemental editing operations for manipulating the shape of a 3D terrain. Their effect on the terrain is as follows:

- Raise: the terrain is raised by augmenting the height values of the points within the area swept by the brush stroke. As this operation adds on the former values, its effect is cumulative.
- Lower: The terrain is lowered within the swept area by decreasing its height values. Since this operation subtracts from the former values, its effect is also cumulative.
- Smooth: It diminishes precipitous shape modifications within the swept area by moving the height values towards the height average within this area. Similar to the prior operation, it overwrites the height values as a result of recurrent applications of this operation on the same area.
- Flatten: The terrain is flattened within the swept area by moving the value of its points to a height provided by the user. The result of this operation is cumulatively destructive, because it eventually overwrites the previous height values as a consequence of recurrent applications of this operation on the same area. Flatten performs as a weighted eraser when the specific height is zero.

### 3.3. 3D Scene Management Component

As mentioned at the beginning of Section 3, the user tool widgets and the terrain mesh are placed inside the 3D scene. These interactable components of the ShAREdT architecture are detailed in the following two subsections.

#### 3.3.1. User Interface

When designing user interfaces of applications for mobile devices, it is necessary to follow a different approach from the one used in desktop user interfaces in order to avoid saturating the screen with multiple widgets like buttons, menus, and windows [33,34]. A common solution is the use of contextual menus, which can be hidden or shown either by the application or the user.

Another option to achieve a proper user interface design is AR. The ShAREdT architecture relies on this technology, since it provides a natural way to make the most of the limited display size of mobile devices. Thus, the screen acts as the workspace, while the digital contents are visualized as part of the 3D scene taken from the real scene by the device’s camera.

AR-based applications run on a single window in full-screen mode (per user), but it that usually does not provide widgets, such as borders, scrollbars, or menus. If buttons, sliders, or labels are required, these widgets are normally displayed in a different layer over the digital content with the aim of keeping them perceptible to the user at all times. However, a distinct approach to the user interface design has been followed by embedding a minimum of widgets along with the terrain mesh in the 3D scene, using an uncluttered and simple layout.

**Figure 7:**
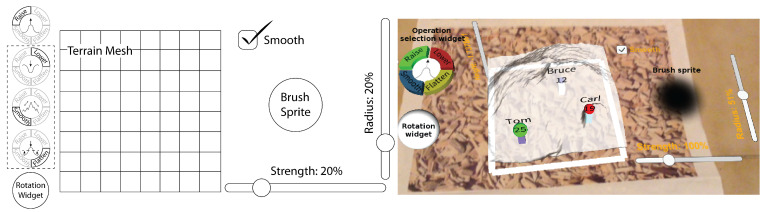
User interface of the ShAREdT-based collaborative application for terrain sketching, where avatars on the terrain mesh denote the identity, location, and current action of participants in a collaborative session.

Figure 7 shows the user interface of the collaborative application developed to validate the ShAREdT architecture. The terrain mesh is shown at the center of the 3D scene. In the previous version of our collaborative application for terrain sketching [27,28,29], we observed that users searched for a strategy to rotate the terrain mesh, in addition to walking around the physical marker. Therefore, as shown in Figure 7, we added a rotation widget that acts as a trackball, which spins the terrain mesh around two axes. This widget is also available when the user interacts with the application in standard mode. The user can rely on the rotation widget to visualize its shape from any perspective, together with an extra-slider to zoom in/out the terrain mesh.

Besides the interactable terrain mesh and rotation widget, Figure 7 shows an operation selection widget and a brush sprite, which allow collaborators to modify the terrain mesh (see Section 3.3.2).

This minimalist user interface approach has three main advantages:

1. The user does not have to call a hidden menu every time he needs to perform an action;
2. The available space to put widgets is potentially larger than the screen size; and
3. The widgets are virtually accessible at any given moment; they are not invariably shown, but just when the user fixes the camera on the area where these widgets are envisioned to be (This interaction can be seen on https://computacion.cs.cinvestav.mx/~lmsanchez/images/MDPI.mp4 (accessed on 10 November 2021)).

#### 3.3.2. Terrain Direct Manipulation

To directly manipulate a terrain mesh, the ShAREdT architecture defines the four operations described in Section 3.2.2. Each collaborator can select one of these operations by tapping on the equivalent button in the operation selection widget (see Figure 7). Just one operation can be actived at a time. Therefore, if the user chooses another operation, the former one is systematically deactivited.

The four possible states of the operation selection widget are shown at the leftmost side of Figure 7. When an operation is selected, the corresponding button is accentuated, while its icon is displayed at the central part of the widget. These four icons are designed with the aim of clearly conveying the operation’s effect that is being applied on the terrain mesh by the ongoing tool.

When an operation is performed on the terrain mesh, each collaborator uses a digital brush to *depict* the areas where he aims at modifying. As mentioned before, a *sprite* shaped like a brush has been placed to the right of the terrain mesh, in order to help each collaborator have an accurate idea of the terrain area that will be touched when drawing on it (see Figure 7).

A brush can be shaped as a square, triangle, or circle. For the moment, two basic circular brushes are provided: a soft brush and a hard brush (the former is shown at the rightmost side of Figure 7). To switch between these two brushes, the user has two options, either by double-tapping on the brush sprite or by using the *Smooth* checkbox (if it is checked, the soft brush is activated, else the brush is hard).

The effect of an editing operation is determined by the following brush properties: strength, shape, and radius. Each collaborator can change the stroke strength and brush radius by making use of the sliders shown in Figure 7. A visual feedback of the brush radius and stroke strength values are respectively given by the sprite-size and sprite-transparency (the more transparency, the less strenght).

Each editing operation is performed within a circle, whose radius is the one of the brush sprite and whose center is provided by the user with a touch on the terrain mesh. The amount of modifications made on each point within the circle is determined by the brush type (i.e., soft or hard) and a strength factor given by the user through a slider; the brush shape masks the effect of an editing operation on the terrain mesh. When a brush stroke is made on the screen, the series of touch events takes place at different coordinates and makes consecutive changes in the terrain mesh, whose effect is the edition along the touch gesture path.

Concerning the *Flatten* operation, each user has to give the height value. To fix this property, the 3D scene provides a slider that displaces an indicator plane to the chosen height, with the goal of giving the user visual feedback. This slider is placed parallel to the vertical axis of the screen and is only visible when the *Flatten* operation is activated.

### 3.4. Group Awareness Component

The *Group Awareness* component of the ShAREdT architecture provides collaborators with three elements of awareness information about others: identity, location, and current action [35]. In the 3D scene, these elements are displayed as avatars on the terrain mesh. These avatars are represented as little boxes that show the user’s name, where they are (i.e., the last position where their finger was on the terrain mesh), and what action they are performing (e.g., raise the terrain mesh).

To manage awareness information, *Group Awareness* relies on the *Data Sharing* component, which provides state information about all active participants in a collaborative session. State information is represented by the *Collaborators State* data component in Figure 2. Besides each collaborator’s name and current editing operation, this data component stores the properties defining the brush each user applies on the terrain mesh (e.g., strength, shape, and radius) as well as the height value needed by the *Flatten* operation.

For each present collaborator, the *Group Awareness* component draws an *Avatar* on the 3D scene (see Figure 2). An avatar takes the form of a little box that is situated in the last place where the corresponding collaborator *painted* on the terrain mesh. Each box has a random but unique color, as well as a label associated with the collaborator’s name, and a sphere that denotes the last state change in the collaborator’s brush.

Figure 7 shows three participants in a collaborative session: Tom, Bruce, and Carl, whose avatars are respectively represented by lilac, white, and mint boxes. The sphere color corresponds to the editing operation button chosen from the operation selection widget (i.e., green for *Raise*, red for *Lower*, blue for *Smooth*, and yellow for *Flatten*). For instance, Figure 7 shows that the operations *Raise*, *Smooth*, and *Lower* were respectively applied by Tom, Bruce, and Carl on the terrain mesh. The sphere size indicates the brush size, which is also shown in pixels (1–50) at the center of the sphere. Other properties, such as brush smoothness and strength, or the height needed by the *Flatten* operation, are not shown to avoid saturating the 3D scene. However, these properties can be deduced by collaborators from their effect on the terrain mesh, e.g., if the terrain mesh quickly raises, the strength is substantial.

Neither the color nor the position of each box needs to be stored in the *Collaborators State*, since the color is selected when the avatar is created and does not change for the duration of a collaborative session. The box position is modified when *Group Awareness* receives a message from *Data Sharing* about the editing operation that was just performed by the corresponding collaborator.

### 3.5. Data Sharing Component

**Figure 8:**
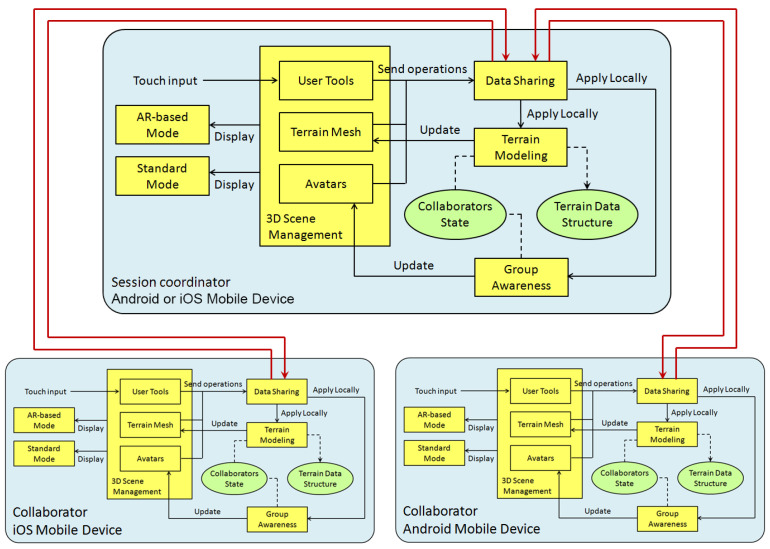
Distribution and communication schemes for the ShAREdT-based collaborative applications for terrain sketching.

The target settings we are interested in have to do with the *face to face or co-located collaboration* taxonomy in the space-time matrix by Ellis et al. [36], which designates a group of collaborators that work together at the same time and in the same place. To allow co-located collaborators to create and modify a terrain mesh in a concurrent way, the ShAREdT architecture follows a replicated scheme, meaning that each application instance is formed by all data and functional components introduced at the beginning of Section 3. Figure 8 shows three application instances (each one represented by a box), which are connected by one-way communication channels (each one denoted by an arrow).

To connect *m* application instances, one for each participant in a collaborative session, the ShAREdT architecture might use a peer-to-peer model, in which each application instance can establish two one-way communication channels with another. Nevertheless, rather than creating m(m−1) channels to connect every pair of application instances, just 2(m−1) channels are established supposing that, in face to face collaboration settings, all application instances are connected through the same WiFi network. Therefore, not only the network traffic can be decreased, but also the data exchange process can be simplified.

The communication support provided by the ShAREdT architecture follows a coordination-based multiplatform model [35], where one of the application instances plays the role of *session coordinator*, which manages connections and interactions among all participants in a collaborative session. For example, in Figure 8, the application instance acting as the session coordinator is hosted by the mobile device of one the collaborators. This mobile device can be Android or iOS. The other application instances can also run on heterogeneous mobile devices. It is important to mention that any application instance can be the session coordinator, and the user interface is the same for all collaborators, so there is not a particular one for the session coordinator.

If a modification occurs on the data components (see *Collaborators State* and *Terrain Data Structure* in Figure 8), state change or editing operation messages (instead of the whole collaborators state or the whole terrain mesh) are transmitted by the *Data Sharing* component. Thus, the network is not saturated with unnecessary information.

When an application instance issues a state change or editing operation message, this instance first sends the message to the session coordinator, which distributes it towards all connected instances, including the one that initially issued the message. When this instance receives its own message, the corresponding component (*Group Awareness* or *Terrain Modeling* in Figure 8) then processes it locally. In this way, the application instance that issued a message is prevented from processing it twice. The other instances also process the message in the same way as soon as they receive it from the session coordinator. According to this data updating approach, every application instance receives its own messages as if they would have been sent from another instance. This design decision simplifies the data updating process, since all messages are handled in the same way, without dividing into the own state and the state of others. In the following two subsections, the updating mechanisms for the data components of our architecture is detailed.

#### 3.5.1. Collaborators State Updating

At the beginning of a collaborative session, every application instance starts with an empty *Collaborators State* data component, which is initialized in the following way. When a collaborator logs in to the application, the corresponding avatar (see Section 4.1) issues a state change message, which is forwarded by the *Data Sharing* component to its pair hosted by the session coordinator. In turn, this one broadcasts the state change message to all connected instances, including the one that initially sent this message. Upon receiving it, the *Data Sharing* component, in each application instance, forwards this message to the local *Group Awareness* component, which verifies whether there exists an entry in *Collaborators State* with the collaborator identifier included in such a message. If it does not exist, *Group Awareness* creates a new entry in *Collaborators State* using that identifier and initializes this new state with default values. In case an entry already exists, *Group Awareness* resets this state to default values.

When a collaborator selects a new editing operation or modifies the brush properties, the corresponding user tool widget (see Section 4.2) sends a state change message to the *Data Sharing* component of the local instance. Then, this component forwards the message to its pair hosted in the session coordinator, which transmits it to all connected instances. When this state change message arrives at an application instance, the *Data Sharing* component eventually forwards it to *Group Awareness*, which searches for the participant’s identifier in *Collaborators State* to update the corresponding information.

#### 3.5.2. Terrain Data Structure Updating

Each application instance maintains a local replica of the terrain data structure. When an editing operation is performed by a collaborator, while interacting with the terrain mesh widget (see Section 4.3), this one issues an editing operation message, which is sent by the *Data Sharing* component to its pair hosted in the session coordinator. In turn, this one broadcasts the message to other connected instances, which process it to update their own local replicas, in the following way. When an application instance receives an editing operation message, *Data Sharing* informs the *Terrain Modeling* and *Group Awareness* components of this message. First, *Terrain Modeling* searches for the participant’s editing operation and brush properties in *Collaborators State*, using the identifier contained in the message. Then, *Terrain Modeling* applies this editing operation in the position *x*, *y* of *Terrain Data Structure*, using such brush properties, while *Group Awareness* updates the position of the collaborator’s avatar in *Terrain Mesh*. Although consistency mechanisms have not been implemented for the moment, in most cases the operations are executed in the order in which they were sent to all instances (in our tests we only noticed minor inconsistencies thanks to the fact that they were performed on a local area network).

In distributed systems, there exist many concurrency control mechanisms, but in collaborative systems, this problem is solved in a different way, since these systems need to consider both interactions among computers and interactions among people. For each application domain, a trade-off should be found between the response level perceived by collaborators and the optimism level of the concurrency control mechanism used in the collaborative application. In some cases, tight control might be even unnecessary. The mere idea of allowing a collaborative application to lack a concurrency control mechanism might seem unacceptable but, according to Greenberg and Marwood [37], it can be a reasonable strategy. Depending on the type of application (in this case, a terrain sketching application for co-located collaborators with mobile devices) either inconsistencies might be irrelevant, or collaborators could mediate their own actions and solve their conflicts. A small amount of accumulated inconsistencies can be acceptable to collaborators, or unperceived. For instance, in sketching applications, the importance lies in discussing ideas and creating a design concept rather than generating a final product.

## 4. Implementation of a ShAREdT-Based Collaborative Application

In this section, we expose some implementation details about the improved version of our application, which has been developed using Unity 3D [38], a game engine commonly employed to develop many kinds of applications besides games. Unity offers a wide range of useful utilities to create multiplatform 2D and 3D applications for desktop computers running Windows, MacOS, or GNU/Linux, and also on mobile devices running iOS or Android.

We have also used Vuforia [39], a development library that facilitates the incorporation of AR functionalities in Unity-based applications, Android, and iOS. Vuforia relies on computer vision algorithms to identify a prior learned marker and to augment virtual objects placed parallel to it.

Our prototype is built inside the Unity engine. All objects presented in the 3D scene are GameObject instances, since this class owns all essential attributes to situate an object in the 3D scene, e.g., scale, orientation, and position. This class is also related to the Mesh class, which contains the mesh data, e.g., faces, material, texture, and vertices, to depict the object on the screen. In addition, Unity defines the MeshCollider class to detect whether the object is hit by a touch gesture and on which point. It is possible to associate other classes with GameObject, with the goal of defining new behaviors according to the application’s requirements.

Next, three kinds of widgets for the 3D scene are described.

### 4.1. Avatars Widgets

**Figure 9:**
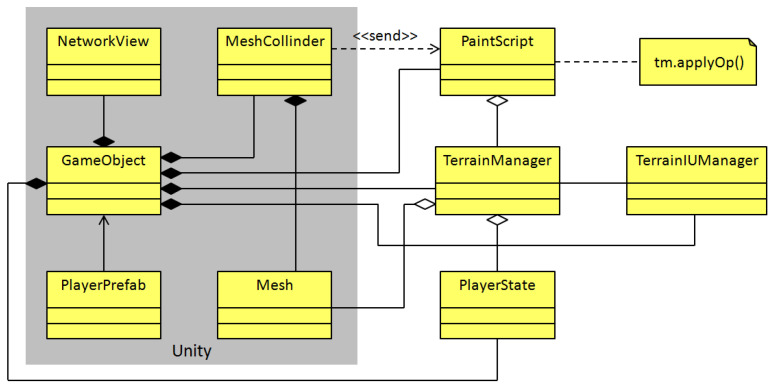
Class diagram of the collaborator creation and terrain mesh sketching.

In collaborative applications, it is needed to stay informed about other participants’ state during a working session. As mentioned in Section 3.4, tiny avatars are used to exhibit the touches performed by collaborators. Such avatars are GameObject instances, and they are also related to the PlayerState class, which contains the collaborator’s name, as well as other attributes that specify the chosen editing operation and brush shape, as shown in Figure 9. The PlayerState methods facilitates the creation and resizing of a dynamic array (named mask) that serves as a digital brush, whose properties change in real-time.

When a participant joins a collaborative session, the Unity Prefab mechanism is used to create GameObject clones. In this case, the PlayerPrefab class is defined as a template in the creation of collaborator avatar instances.

In Unity, it is feasible to develop multi-user applications employing the NetworkView class, which has an association with GameObject. Through NetworkView, each GameObject instance can automatically update its translation, rotation, and scale properties and perform RPCs on all connected application instances. This mechanism is used by the collaborator avatars to update their location and PlayerState instance.

Besides the collaborator avatars, there are other GameObject instances that compose the 3D scene: the user tools and terrain mesh widgets. Behavior classes are attached to those GameObject instances, in order to handle their reaction to user touches.

### 4.2. User Tools Widgets

The user tools of our collaborative application contains a checkbox, a rotation widget, a sprite, three sliders, and four buttons (see Figure 7). These widgets are also implemented as GameObject instances, but rather than associating a behavior class with each widget, the TerrainUIManager class was created with the aim of managing them (see Figure 9).

As for the checkbox and the three sliders, they are compliant with the common functionality programmed by Unity developers for this type of widgets. When a collaborator interacts with these widgets, the corresponding TerrainUIManager methods are invoked to update the PlayerState property for that collaborator, i.e., the brush strength, smoothness, and radius, as well as the value for the *Flatten* operation.

The four buttons are GameObject instances. With the aim of tracking each time a collaborator taps on these buttons, the *tap gesture* behavior from TouchScript (TouchScript is a multitouch framework for Unity, designed to receive touch events from different sources and to facilitate the handling of gesture interactions. It can be found in the Unity Asset Store) has been added to them. A sphere shape is used to implement the rotation widget, and the *pan gesture* behavior has been attached to it, in order to track the dragging action on the sphere. This action causes the terrain mesh rotation.

The sprite is a GameObject instance, whose Mesh instance is simply a plane associated with a pair of transparent textures to show the hard and smooth brushes. The *double tap gesture* and *pinch gesture* behaviors have been added to this plane to invoke the TerrainUIManager methods that modify the brush radius and smoothness. For simplification, all these behaviors are not shown in Figure 9.

### 4.3. Terrain Mesh Widgets

According to Figure 9, the terrain representation in the 3D scene is a GameObject instance, which is associated with the MeshCollider class that handles the collision of touch events with the Mesh instance of the 3D terrain (implemented as a GameObject instance).

The PaintScript class has been added to handle the reactions of the terrain mesh to touches; it is also responsible for calculating the right index in the terrain array from the touch coordinates. PaintScript obtains touch events from MeshCollider and performs operations via the TerrainManager class. This one contains the terrain array and applies editing operations on it, using any collaborator state included in an array of PlayerState instances. In addition, TerrainManager has a reference to the Mesh instance of the terrain and maintains the Z coordinate values of its vertices up-to-date, thanks to the data stored in the terrain array; therefore, the terrain shape is modified in real-time.

The terrain mesh, as a GameObject instance, also relies on the NetworkView class to trigger editing operations through RPCs, invoking the applyOp() method with the precise PlayerState instance in all connected application instances.

## 5. Tests and Results

In this section, we explain the tests conducted with several groups of users who conjointly sketched 3D surfaces using two tools: (1) our ShAREdT-based collaborative application running on mobile devices, and (2) a free web browser desktop application called WebGL terrain editor [14], which is neither AR nor collaborative but offers brush-based operations that are akin to our prototype. We selected WebGL terrain editor because, first of all, it is suitable for novice users, as it offers minimal and straightforward widgets. Second, the way to build and manipulate terrains is similar to our prototype. Finally, WebGL is a free and available application.

The purpose of the tests described below is to compare the usability and user experience: (1) between the WebGL terrain editor and the ShAREdT-based application in both the standard and AR-based interaction modes, and (2) between these two forms of interaction provided by our prototype.

**Figure 10:**
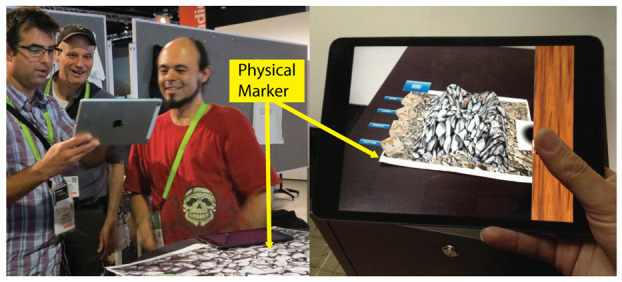
A snapshot of some participants of our tests.

Participants were MSc and PhD students at the Computer Science Department of CINVESTAV-IPN. This was the first 3D modeling experience for the majority of them (see Figure 10).

### 5.1. Test Management

The next assumptions were made to carry out our tests:

- There is a relatively small group of collaborators in the same room wanting to create a 3D surface for a certain purpose.
- They have a desktop computer with an internet connection and a web browser.
- Each of them has a WiFi-enabled mobile device running iOS or Android (besides to the Apple devices used in the former tests, the following two types of Android devices were also used in these tests: (1) Samsung Galaxy S6. Screen size: 5.1 inches. Weight: 138 g. Camera: 16 MP and (2) Samsung Galaxy Tab S. Screen size: 10.5 inches. Weight: 465 g. Camera: 8 MP).
- They have a working WiFi network and at least one mobile device with a public IP address.
- There is no packet loss in the exchange of short messages.

To start a collaborative session in the ShAREdT-based application, the device with a public IP has to execute its instance of the application, which will also play the role of session coordinator. The collaborator of this device should provide a unique nickname and tap the *Start Session* button at startup. This collaborator can also edit the terrain mesh as well as other participants, but this device should not leave the session until the end.

Each participant must connect his device to the WiFi network and launch the application. They also have to provide a unique nickname and tap the *Join to Session* button. Then, they must choose, from the list of available sessions, the one started by the collaborator whose instance of the application acts as session coordinator.

Once everyone is connected, the editing phase can start. At the beginning of the collaborative session, all participants have the terrain mesh and the editing tools exactly in the same state. To guarantee this initial state, users can tap the *Reset* button. Then, they can modify the terrain mesh using the editing tools without further restrictions. At the end of the session, participants can tap the *Leave* button to abandon the session. In the current version of the application, the issue of latecomers [40] has not yet been addressed, thus it is assumed that participants will not reach the collaborative session lately.

In principle, the ShAREdT-based application can support an arbitrary number of participants. However, during the test sessions, we observed that the main limiting factor was the bandwidth available on the local WiFi network. For instance, using a public WiFi network, our ShAREdT-based application was able to support up to 13 connected participants before starting to lose messages or saturating the network. These problems occured depending on the time of the day, suggesting that they were not caused by the prototype implementation, but by the network public character. To avoid them, a mobile zone (by means of this functionality, a mobile device plays the role of WiFi access point, to which other devices can connect, constituting an ad-hoc network that can be declared as public or private) was also used on an Android device, allowing up to 10 participants to establish a connection. Let us notice that this limitation is due to the hardware. Using this ad-hoc WiFi network solution, a good response was obtained from the application, thanks to the private network.

To evaluate the user experience of the tested applications, we relied on the AttrakDiff tool [41], whereby users assign a weight to pairs of opposite words, indicating how much an application can be described with one or another word. Tests were conducted with 20 participants (the maximum number of users supported by AttrakDiff) who formed five groups of four individuals each.

On the other hand, the WebGL terrain editor was used on a desktop computer, since both mouse buttons are needed to manipulate the 3D model. This editor is not designed for a touch screen. Thus, each group of collaborators has a PC to interact with WebGL using a turn-taking policy.

Three tests have been carried out with each group of users. Before starting the tests, participants used, for some minutes, the WebGL terrain editor and the ShAREdT-based application in both standard and AR modes in order to get acquainted with them. In the first test, participants were asked to model a conical shape with a crater at the summit (similar to a volcano) using WebGL and ShAREdT in standard mode. In the second test, they were asked to model the “M” letter with WebGL and ShAREdT in AR mode. Finally, in the third test, they were asked to model the “Q” letter using ShAREdT in standard and AR modes. These three tasks were selected because they have relatively simple shapes and a similar difficulty, but they are different enough to avoid the undesirable learning effect.

At the end of each test, every user evaluated each application with the AttrakDiff tool. We obtained a total of 120 questionnaires, which consisted of two per participant in each test.

### 5.2. Results

**Figure 11:**
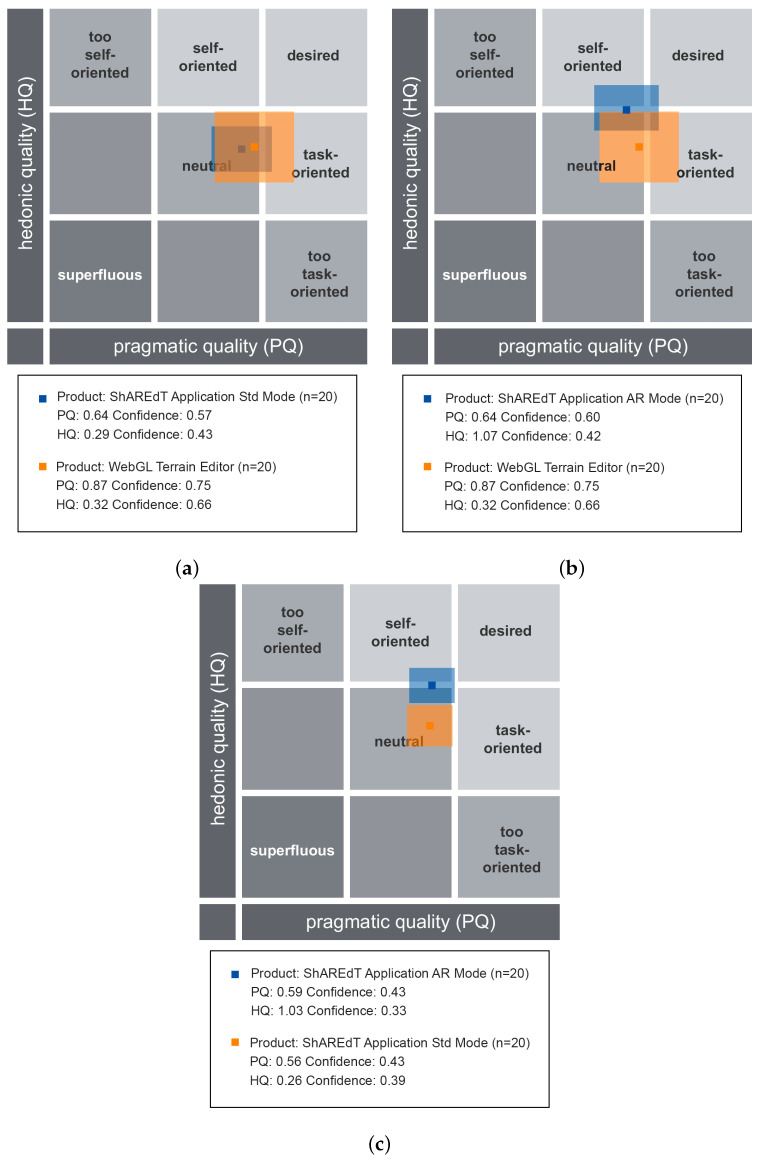
AttrakDiff porfolio charts for the three comparisons. (**a**) ShAREdT in standard mode vs. WebGL; (**b**) ShAREdT in AR mode vs. WebGL; (**c**) Standard mode vs. AR mode of ShAREdT.

**Figure 12:**
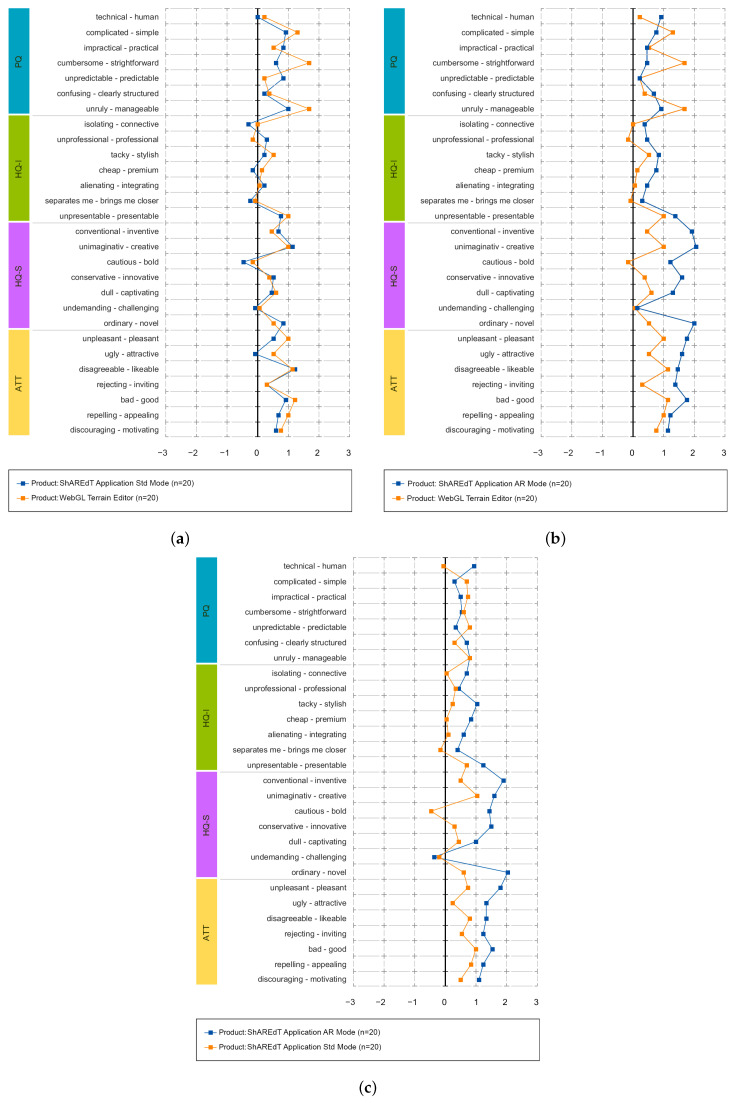
Semantic pairs for the three comparisons. (**a**) ShAREdT in standard mode vs. WebGL; (**b**) ShAREdT in AR mode vs. WebGL; (**c**) Standard mode vs. AR mode of ShAREdT.

The results of these comparisons are shown in Figure 11 and Figure 12. In these tests, the modeling abilities of participants were not assessed, but rather their perception of each application, using just one type of questionnaires. The following dimensions were evaluated [41]:

- *Pragmatic Quality (PQ)*: It refers to the usability of an application and denotes how well users are in reaching their objectives through the application.
- *Hedonic Quality Identity (HQ-I)*: It shows to what extent the application allows the user to relate to it.
- *Hedonic Quality Stimulation (HQ-S)*: Humans have an innate need to advance and create. This dimension denotes to what extent the application is able to support this requirement in terms of stimulation, interesting and novel functions, interaction, presentation styles, and contents.
- *Attractiveness (ATT)*: It represents a global value of the application subject to the quality perception.

AttrakDiff provides three types of graphs for each test. Figure 11 shows the portfolio charts, where the values of the *hedonic quality* (HQ) are depicted on the vertical axis (the higher the better). The horizontal axis denotes the values of the *pragmatic quality* (PQ) (the further to the right the better). The graph domain is divided in nine regions, some of them are labeled with categories, such as *neutral*, *desired*, and *superfluous*, whereby the applications are graded. Based on the values of HQ and PQ, the examined applications may be situated in one or more “character-regions”.

In Figure 11a, the WebGL terrain editor is compared with the ShAREdT-based application in standard mode. The two colored rectangles denote the results of these applications. The coordinates of the center in each rectangle indicate the average obtained in both qualities, and the rectangle size represents the confidence intervals. A bigger rectagle means that weights assigned by users were more varied than in the case of a smaller rectangle. The orange rectangle (the biggest one) corresponds to the WebGL terrain editor. The blue rectangle (the smallest one) shows the results of ShAREdT in standard mode. Both applications were perceived as *neutral* and their respective centers are very close, though they slightly tend to the *desired* region. In the case of WebGL, users show a larger disagreement in both qualities.

Figure 11b shows the results of comparing WebGL (orange rectangle) with ShAREdT in AR mode (blue rectangle). Users perceived both applications as *neutral* with a similar *pragmatic quality* (PQ), but the use of AR had a positive impact on the *hedonic quality* (HQ), which practically places our prototype on the boundary of the *neutral* region. Once again, users show a larger disagreement with WebGL than with ShAREdT.

Figure 11c shows the comparison between the standard (orange rectagle) and AR (blue rectangle) modes of our prototype. Let us notice that users’ perception in the *pragmatic quality* (PQ) is very similar for both interaction modes, but a significative improvement in the *hedonic quality* (HQ) for the AR mode can be observed.

**Figure 13:**
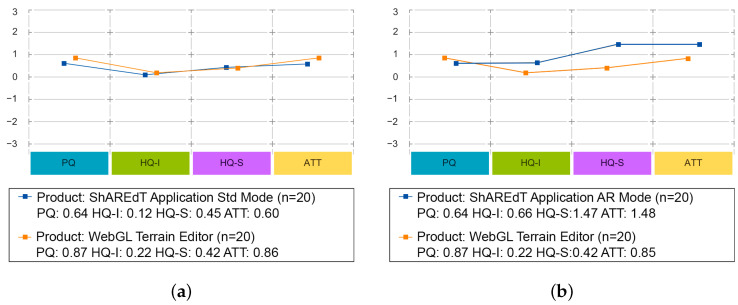

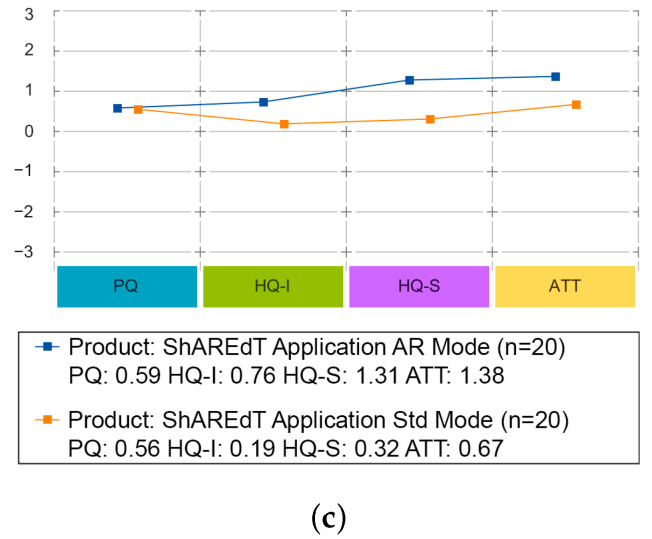
Dimensions charts for the three comparisons. (**a**) ShAREdT in standard mode vs. WebGL; (**b**) ShAREdT in AR mode vs. WebGL; (**c**) Standard mode vs. AR mode of ShAREdT.

Figure 13 features charts of average values for the *pragmatic quality* (PQ), *hedonic quality identity* (HQ-I), *hedonic quality stimulation* (HQ-S), and *attractiveness* (ATT) dimensions. These kind of graphs has a range that varies from −3 to 3, denoting the scale of seven steps that each semantic differential has (lower is worse). Figure 13a shows that WebGL and ShAREdT in standard mode mainly received similar neutral evaluations, since the values in all dimensions are close to zero, but WebGL has a very slight advantage. In Figure 13b, ShAREdT in AR mode obtained more favorable results than WebGL, which was just slightly better in PQ. Figure 13c shows that ShAREdT was better evaluated in AR mode than in standard mode, and they almost tied in PQ.

Finally, Figure 12 presents the average values for each pair of words in the questionnaire. In Figure 12a, the evaluation of semantic differentials was similar in almost all cases, and the only noticeable differences were “cumbersome-strightforward” and “ugly-attractive”. Figure 12b shows that, in most pairs, ShAREdT in AR mode was superior to WebGL, which just had notable advantages in three semantic differentials: “complicated-simple”, “cumbersome- strightforward”, and “unruly-manageable”. In Figure 12c, ShAREdT in AR mode was again better evaluated than the standard mode. Only in the *pragmatic quality* (PQ) semantic differentials did ShAREdT, in standard mode, have a slight advantage.

## 6. Discussion

To discuss our test results, it is necessary to briefly return to the scenario that we presented at the beginning of this paper: a group of colleagues who can express their ideas casually to achieve a basic 3D terrain that later, if they wish, they can edit in another more sophisticated piece of software. Under this premise, we created a simple application that allows novice users to make basic 3D terrain sketches, with the added value of being collaborative and offering an AR interaction mode to facilitate work.

We sought to compare the ShAREdT application with another similar tool, a basic design application for beginners, and usable in casual environments. this ruled out professional tools such as Maya or Blender. At this point, we realized that there is a lack of applications with these characteristics, and it was not until recent years, thanks to the power of mobile SoCs and the integration of technologies such as LiDAR, that 3D modeling applications have appeared for mobile devices [42,43]. Therefore, we chose WebGL Terrain Editor as a comparison application, even though it is not mobile, collaborative, or AR capable. So why did we choose it? As we already mentioned, to our knowledge, there are no applications that meet all of these characteristics and that are also aimed at novice users. Thus, if a professional tool had been used, a critical bias would have been created; participants would probably have found it very confusing, which would have led them to have a negative experience, creating unreliable results.

However, having taken WebGL as a comparison application allows our results to have a context beyond evaluating our prototype and to provide more evidence for other related research. For instance, contrast 3D modeling solutions on platforms other than a PC [44], AR’s contribution in these types of tools, and the elements of collaboration in developments of a similar nature [45,46].

Regarding the difference in platforms, we can say that we obtained a fascinating result since, as can be seen in Figure 11 and Figure 12, participants perceived the standard mode of ShAREdT and WebGL in a very similar way. It must be considered that our participants had little or no experience in 3D modeling in such a way that they did not have preconceptions or prejudices that favored a specific platform. Due to their background as postgraduate students who carry out all their professional activities on a PC, one might think *a priori* that this mode of interaction had a particular advantage, but the results make it clear that this was not the case; for them, the experiences of using a mobile device and a PC were the same. This is important for interpreting the rest of the results, since we consider it a baseline indicating an insignificant platform difference.

On the other hand, we have the AR aspect. In comparing the AR mode of ShAREdT against WebGL and the standard mode, we can see that participants better received the AR mode. Looking at Figure 11, we see that the *pragmatic quality* (PQ) was very similar in the three tests, but the *hedonic quality* (HQ) was slightly higher in the tests involving ShAREdT in AR mode. This tells us that users could perform their tasks equally well in all cases, but they found AR more enjoyable. These results follow the trends that can be found in the state of the art, e.g., Fechter et al. [47] mention that virtual reality can facilitate tasks close to real-life activities. Although in our case it did not involve tasks of this nature, the results indicate that AR could have a beneficial effect for users with little experience to have more pleasant experiences in 3D modeling.

Finally, we will address the collaborative aspect. Collaboration was a latent variable in our study. Therefore, it cannot be directly observed in the results but must be seen as part of the entire evaluation. Our application can lead to confusing situations from what we observed in the tests and from the participants’ statements. This is not strange, since it is well known that an excellent collaborative tool is founded on elements of coherence, awareness, shared space, and concurrent work [48,49]. Of course, 3D modeling tools are no exception. However, this confusion was not significant enough to harm the participants’ perception, since the most relevant point is that it allowed them to express themselves and implement their ideas.

An important aspect that must be highlighted is the choice of AttrakDiff as an evaluation tool. Our proposal does not intend to contribute to Computer Vision or Groupware formally but is a demonstration of how elements of these areas can help create solutions to facilitate the expression of people, in this particular case, in the modeling of 3D terrains. Moreover, it is precisely because of this expression that we decided to evaluate with a user experience tool, since it allows us to measure, in a quantitative way, at the same time, the pragmatic and hedonic perceptions of the users. Beyond performance measures or formal verification, we were interested in whether the components proposed in our architecture could create a valuable product for users.

### 6.1. Limitations

Some important limitations of the ShAREdT architecture are:

- The use of markers affects people’s mobility, since their devices are physically bound to them. To use markers, collaborators need a physical space to move around and must not lose them, since the AR mode of ShAREdT becomes unusable.
- The current version of our architecture does not provide any support for latecomers nor for asynchronous work. Thus, collaborators are not free to join the session late or at different moments, since the current proposal lacks mechanisms to manage persistent collaborative sessions (e.g., logs of past actions on the 3D scene).
- The editing functionality is basic. More elaborate operations and a broader diversity of brush shapes would allow users to sketch more sophisticated terrains.

### 6.2. Future Work and Implication

This work establishes a broad set of possibilities for further improvement of the ShAREdT architecture. As it only provides support for face to face collaboration settings, in the near future we plan to support distributed collaborative work so that people are not limited to work in the same physical place. However, this additional functionality needs the redesign of some functional and data components or the creation of new ones:

- For sketching applications, in which the goal of collaborators is to create preliminar approaches rather than final models of 3D terrains, minor errors are acceptable, as found by Greenberg and Marwood [37] in their work on 2D editing tools. These observations agree with ours, since differences among the terrain meshes from the several application instances were considered as irrelevant by our users. In both studies, errors can be negligible because they happen sporadically, thanks to the local area network in which those tests were made. However, in wide area networks, the rate of inconsistencies increases significantly [19], so data coherence has to be ensured using suitable mechanisms, such as operational transformation [18], which transforms editing operations before applying them on the terrain mesh.
- At the user interface level, collaborators would need additional awareness information to coordinate their work. However, adding new awareness widgets in each user’s view of the 3D space is not a good idea, since the view size is limited to the screen size of the mobile device in the standard mode of ShAREdT. An alternative solution could be the creation of a multi-device environment for each collaborator, so that the new awareness widgets can be displayed on another surface. Nevertheless, collaborators would face problems to interact with an additional device, since one of their hands is used to hold the mobile device and the other to interact with the 3D modeling application through screen touches. Consequently, multi-modal interaction would be needed in this case, e.g., voice or gestures, to interact with other devices. Besides, an audio or video support might be useful to communicate with others.
  The communication scheme of the ShAREdT architecture also needs modifications. In particular, the centralized session coordinator, which is in charge of the broadcasting state and editing operation messages among all application instances, must be replaced by a peer to peer communication approach or a distributed session coordinator. In this way, collaborative editing sessions would be fault-tolerant.

## 7. Conclusions

The main contribution of this work is ShAREdT, a new architecture for facilitating the development of terrain sketching applications for face-to-face collaboration settings. The applications developed with the ShAREdT architecture’s guidelines are intended for novice collaborators, who can concurrently create and modify 3D terrain sketches using technology on heterogeneous mobile devices without the need for extra equipment, such as stereoscopic viewers, projectors, or HMD.

The ShAREdT application, which served as a proof of concept, implements two interaction modes. The former one is the standard mode, where the application displays the necessary touch widgets so that users can work: an area for the 3D terrain, a brush to create grooves on the terrain (with radius, strength, and smooth controls), a widget rotation that aids in navigation, as well as four tools to sculpt the terrain (raise, lower, smooth and flatten). The latter mode is AR, which has the same widgets and working principles as the standard one, with the particularity that users can have an immersive view of the terrain mesh using their device’s camera and a marker (digital or printed). This enhanced version of the physical world allows users to see the terrain through their device’s camera as if it were a physical object. In both modes, awareness widgets are offered, i.e., users have avatars that inform in what area of the field their colleagues are working and what tool they are using.

To evaluate our application, we compared it against WebGL Terrain Editor, aimed at novice users, and it has basic widgets very similar to ours, even though it is not collaborative, mobile, or AR compatible. We had 20 participants for our tests who compared and evaluated both applications using the AttrakDiff questionnaire, which allowed us to collect the perceived quality of pragmatic and hedonic aspects. From the results obtained, we can conclude that the difference between platforms (mobile and PC) was not significant for the participants. In the same way, the added AR and collaborative features had a positive impact, so participants valued them better when carrying out their tasks. Both features of our application created a more valuable product for end-users.

From a qualitative point of view, we observed that participants employed their dominant hand to draw while holding the device with the other, which is usually more limited. Thus, participants grew tired and changed the mobile device between hands. Moreover, if the participant’s hand is trembling, it will be challenging to draw strokes accurately. This makes sense because, as other studies have observed, AR places a cognitive and physical demand on users, which can cause fatigue and tiredness in the upper extremities [50]. This represents a design challenge, since there is also evidence that AR can be a great collaboration and learning tool, but it has to deal with aspects of the environment and physical effort that it imposes on users [51].

Another exciting behavior observed while testing our application is that participants tended to stand in one spot while creating or modifying a shared terrain rather than roaming around the marker to get diversified viewpoints. Therefore, they did not take full advantage of AR technology. This situation may be due to users not having experience with AR-based applications.

As a final observation, AR can be an outstanding element for inexperienced users to endure 3D modeling applications better. Observing the groups of participants, we could say that four individuals per group make up an excellent collaborative work team. Beyond that, situations of disorder, confusion, and frustration can be created. This becomes a more acute limitation in the AR mode due to the use of markers, since users need ample physical space to move. The standard mode would also have limitations for large groups, as each participant is reflected in a virtual avatar. It could be the case that the 3D space was filled with avatars, which would be very confusing.

Furthermore, large devices, e.g., a 9.7-inch screen, offer a better experience, as users can see more content. However, a larger screen size increases the device’s weight (e.g., a 660 g iPad), so a large device becomes inconvenient if an AR modeling session lasts for a long time. Of course, we need to do more research to understand how these details affect the AR interaction mode.

## Figures and Tables

**Table 1:** Comparative table of the related work and our proposal.

Proposal	Uses AR	Needs Extra Equipment for AR	Is a Groupware	Collaboration Support Type	Application Domain	Allows Concurrent Work	Platform
LandscapAR [15]	Yes	No	No	Not applicable	Terrain creation from white paper	Not applicable	Mobile
ARprism [16]	Yes	Stereoscopic viewer	Yes	Face to Face	Geographical data visualization	No	PC
Product reviewing [20]	Yes	Stereoscopic viewer	Yes	Synchronous-Remote	Reviewing	No	PC
CoMaya [12,13]	No	Not applicable	Yes	Synchronous-Remote	3D modeling	Yes	PC
Second Surface [21]	Yes	No	Yes	Synchronous-Remote	Content creation and sharing	No	Mobile
Clara.io [22]	No	Not applicable	Yes	Synchronous-Remote	Polygonal mesh-based 3D modeling	No	Web
Sandbox [23]	Yes	Kinect and projector	No	Not applicable	Topography model creation	Not applicable	PC
ShowMe [24] RemoteIO [25]	Yes	HMD	Yes	Synchronous-Remote	Training	No	Mobile
